# NCBI BLAST+ integrated into Galaxy

**DOI:** 10.1186/s13742-015-0080-7

**Published:** 2015-08-25

**Authors:** Peter J. A. Cock, John M. Chilton, Björn Grüning, James E. Johnson, Nicola Soranzo

**Affiliations:** 1 Information and Computational Sciences, James Hutton Institute, Invergowrie, Dundee, DD2 5DA Scotland UK; 2 Minnesota Supercomputing Institute, University of Minnesota, 599 Walter Library, 117 Pleasant St. SE, 55455 Minneapolis, MN USA; 3 Department of Computer Science, Albert-Ludwigs-University of Freiburg, Georges-Köhler-Allee 106, Freiburg, 79110 Germany; 4 CRS4, Loc. Piscina Manna, 09010 Pula, CA Italy

**Keywords:** Galaxy, BLAST, Pipeline, Accessibility, Workflow, Reproducibility, Annotation, Sequence analysis

## Abstract

**Background:**

The NCBI BLAST suite has become ubiquitous in modern molecular biology and is used for small tasks such as checking capillary sequencing results of single PCR products, genome annotation or even larger scale pan-genome analyses. For early adopters of the Galaxy web-based biomedical data analysis platform, integrating BLAST into Galaxy was a natural step for sequence comparison workflows.

**Findings:**

The command line NCBI BLAST+ tool suite was wrapped for use within Galaxy. Appropriate datatypes were defined as needed. The integration of the BLAST+ tool suite into Galaxy has the goal of making common BLAST tasks easy and advanced tasks possible.

**Conclusions:**

This project is an informal international collaborative effort, and is deployed and used on Galaxy servers worldwide. Several examples of applications are described here.

## Findings

### Background

The Basic Local Alignment Search Tool (BLAST) [1] has arguably become the best known and most widely used bioinformatics tool in molecular biology. Indeed, BLAST is now so ubiquitous that this term, like PCR (polymerase chain reaction), has become both a noun and a verb in the patois of molecular biology, with the acronym rarely spelt out, and is unfortunately frequently used without citation.

In our opinion, a key factor in the widespread adoption of BLAST has been the easy-to-use NCBI-hosted BLAST web server, which provides (sufficiently) quick search results against regularly updated global sequence databases. The NCBI BLAST web interface is designed for performing one query at a time, which means that larger searches have to be automated for batch processing within a script or by running BLAST as a command line program. Automation also became increasingly important for the analysis of BLAST output as these datasets have grown larger. These needs led to the inclusion in community-developed libraries such as BioPerl [2], Biopython [3], BioJava [4] and BioRuby [5] of code for calling BLAST and parsing its output. Although scripted BLAST workflows greatly facilitated sequence analysis, large-scale BLAST analysis still required a broad bioinformatics skill set, including programming, dealing with complex file types and working at the command line.

With the advent of ‘next generation’ high-throughput sequencing technology, the falling cost of sequence data generation has resulted in a data abundance and all too often analysis bottlenecks. This life science ‘informatics crisis’ was one of the motivations behind the Galaxy Project, which provides a platform for running a broad collection of bioinformatics tools via a consistent web interface [6, 7].

From the Galaxy end-user’s perspective, no local software is required other than a recent web browser, yet the user can run multiple bioinformatics tools (which can be Linux-specific) from their desktop and easily chain together the output of one tool as the input of another. Moreover, Galaxy’s workflow feature enables users to create and share repeatable analysis pipelines. To encourage reproducibility these pipelines can be published as part of the methods in a scientific paper or in a repository such as myExperiment [8].

Galaxy is an open-source project and an international development community has grown up that contributes improvements to the core software and, more importantly, to a growing pool of new tools and datatype definitions that can be added to individual Galaxy servers. These extensions are typically shared via the Galaxy Tool Shed [9], which is a public repository of tools and workflows, from where they can then be installed on individual Galaxy servers. Multiple tools were published in the past 2 years [10–13].

The expansion of a Galaxy developer community outside the project core team has been facilitated by much of Galaxy’s development being coordinated online and in public, using mailing lists, source code repositories (https://github.com/galaxyproject/ hosted by GitHub, Inc.) and project management tools to track issues and feature requests (Trello, hosted by Trello, Inc.). Moreover, the project has been supported by an annual Galaxy Community Conference since 2011 and by full-time staff on the Galaxy Project dedicated to outreach work, which have helped nurture an engaged Galaxy-user community.

Although a free-to-use public server is hosted by the Galaxy Project (https://usegalaxy.org/), many groups and institutes run their own Galaxy servers. Administering a local Galaxy Server enables customization with additional tools of local interest, control of potentially sensitive data and exploitation of local computing infrastructure, or even rented computers from a cloud-computing provider such as Amazon Web Services (AWS) through the use of Galaxy CloudMan [14]. Furthermore, public Galaxy servers are now also being provided by groups wishing to make their own tools immediately available to run by the wider community, thus avoiding the need to write a bespoke web interface [11, 13, 15].

This article describes our NCBI BLAST+ [16] wrappers for Galaxy and associated tools and datatype definitions. Currently, these tools have not been made available at the public server hosted by the Galaxy Project owing to concerns over the resulting computational load (J Taylor, personal communication, 2013). However, they are available from the Galaxy Tool Shed for automated installation into a local Galaxy instance, or from our source code repository (hosted by GitHub, Inc., see Availability and requirements section), and are released under the open-source Massachusetts Institute of Technology (MIT) licence.

### Applications

The NCBI BLAST+ command line Galaxy wrappers and BLAST-related Galaxy tools are listed in Tables 1 and 2, respectively. Table 3 summarizes the datatypes used or defined in Galaxy. We now describe some example cases and workflows in which these tools are combined. Further examples were described in Cock *et al.* [10].

**Table 1:** NCBI BLAST+ Galaxy tools

Galaxy tool name	Description	Reference(s)
NCBI BLAST+ blastp	Protein vs protein	[1, 16]
NCBI BLAST+ blastn	Nucleotide vs nucleotide	[1, 16]
NCBI BLAST+ blastx	Translated nucleotide vs protein	[1, 16]
NCBI BLAST+ tblastn	Protein vs translated nucleotide	[1, 16]
NCBI BLAST+ tblastx	Translated nucleotide vs translated nucleotide	[1, 16]
NCBI BLAST+ makeblastdb	Make BLAST nucleotide or protein database	[1, 16]
NCBI BLAST+ makeprofiledb	Make BLAST protein domain database	[1, 16]
NCBI BLAST+ blastdbcmd entry(s)	Extract sequence(s) from BLAST database	[1, 16]
NCBI BLAST+ blastdbcmd info	Show BLAST database information	[1, 16]
NCBI BLAST+ dustmasker	Nucleotide masking using the DUST algorithm	[1, 16]
NCBI BLAST+ segmasker	Protein masking using the SEG algorithm	[1, 16]
NCBI BLAST+ windowmasker	Window-based sequence masker	[1, 16]
NCBI BLAST+ convert2blastmask	Lowercase masking	[1, 16]
NCBI BLAST+ rpsblast	Protein vs protein domain	[16, 39]
NCBI BLAST+ rpstblastn	Translated nucleotide vs protein domain	[16, 39]

Each row lists a separate Galaxy tool, all available from https://toolshed.g2.bx.psu.edu/view/devteam/ncbi_blast_plus/ on the Galaxy Tool Shed [9]. A separate Galaxy tool is listed for each different underlying NCBI BLAST+ command line tool, except for the blastdbcmd command line tool, whose two main functions are represented as two separate Galaxy tools. We intend to add further wrappers later, including for the command line tools psiblast and deltablast

**Table 2:** Additional Galaxy tools using NCBI BLAST+

Galaxy tool name and URL	Description	Reference(s)
BLAST XML to tabular (https://toolshed.g2.bx.psu.edu/view/devteam/ncbi_blast_plus)	Convert BLAST XML output into tabular output	[10]
BLAST Reciprocal Best Hits (RBH) (https://toolshed.g2.bx.psu.edu/view/peterjc/blast_rbh)	Takes two FASTA inputs, returns table	This paper

Each row lists a separate Galaxy tool, all available from the Galaxy Tool Shed [9]

**Table 3:** Galaxy datatypes used or defined

Galaxy datatype	Type	Description
tabular	Built-in	Tab-separated plain text table, used as default BLAST+ output
text	Built-in	Plain text, used for human-readable BLAST+ output
html	Built-in	Webpage, used for human-readable BLAST+ output with hyperlinks
blastxml	Add-on	BLAST XML output
blastdbn	Add-on	BLAST database of nucleotide sequences, e.g., for BLASTN
blastdbp	Add-on	BLAST database of protein sequences, e.g., for BLASTP
blastdbd	Add-on	BLAST database of protein domain PSSMs, e.g., for RPS-BLAST
maskinfo-asn1	Add-on	BLAST masking information files as text ASN.1
maskinfo-asn1-binary	Add-on	BLAST masking information files as binary ASN.1

Each row lists a separate Galaxy datatype, either available from the Galaxy Tool Shed [9] or already built into Galaxy

#### Assessing a *de novo* assembly

Although more specialized tools exist for the annotation of a *de novo* assembly (e.g., Augustus [17], Glimmer3 [18] and Prokka [19], which we previously wrapped for use in Galaxy [10, 13]), BLAST is often used for a first-pass assessment. The following example is based on a procedure that a local sequencing service, Edinburgh Genomics, had adopted as part of their quality control (later extended as described in [20]).

- Upload or import Illumina reads in FASTQ format.
- Run a fast assembler such as the CLC Assembly Cell (CLC bio, Aarhus, Denmark) which we have wrapped for use within Galaxy to generate an initial set of contigs [21].
- Compare these initial contigs to the NCBI non-redundant protein sequence database (NCBI NR) using BLASTX, requesting at most one hit and tabular output including the taxonomy fields (and optionally the hit description).

As the CLC Assembly Cell software is proprietary, our exemplar workflow, available from the Galaxy Tool Shed [22] and myExperiment [23], starts from a previously generated or imported transcriptome assembly. This workflow analyses a sample of 1000 sequences only and uses Galaxy data manipulation tools to produce a sorted tally table of species hits suitable for visualization within Galaxy as a pie chart.

This simple taxon assignment can detect obvious contamination or sample mix-up. However, this kind of simple ‘Top BLAST hit’ analysis should be treated with caution owing to the potential for spurious matches, or matches to misannotated sequences, such as contaminants, in published whole-genome shotgun assemblies (see, for example, Yong [24] and references therein).

#### Finding genes of interest in a *de novo* assembly

As sequencing costs have fallen, for many organisms it is now practical to sequence the entire genome when interested primarily in a single gene family. In this situation, BLAST might be used within Galaxy as follows:

- Upload or import the (meta-) genome or transcriptome assembly in FASTA format.
- Upload protein (or nucleotide) sequence of the gene(s) of interest.
- Run the makeblastdb wrapper to create a BLAST nucleotide database from the assembly.
- Run the blastx (or blastn) wrapper using the gene(s) of interest as the query against the new database.
- Filter the matching contigs from the assembly FASTA using the “Filter sequences by ID” tool [10, 25] (or similar).

If required, rather than extracting complete contigs, Galaxy has tools for working with genomic intervals that could be used to select the matched regions only, as in the next example.

#### Identifying candidate gene clusters

Identification and analysis of gene clusters is an important task in synthetic biology [26, 27]. Unfortunately, identifying candidate gene clusters is complex and can take hours for a single genome. However, with prior knowledge about the expected genes in a cluster, the genome can be screened in a way that limits the search space dramatically.

**Fig. 1:**
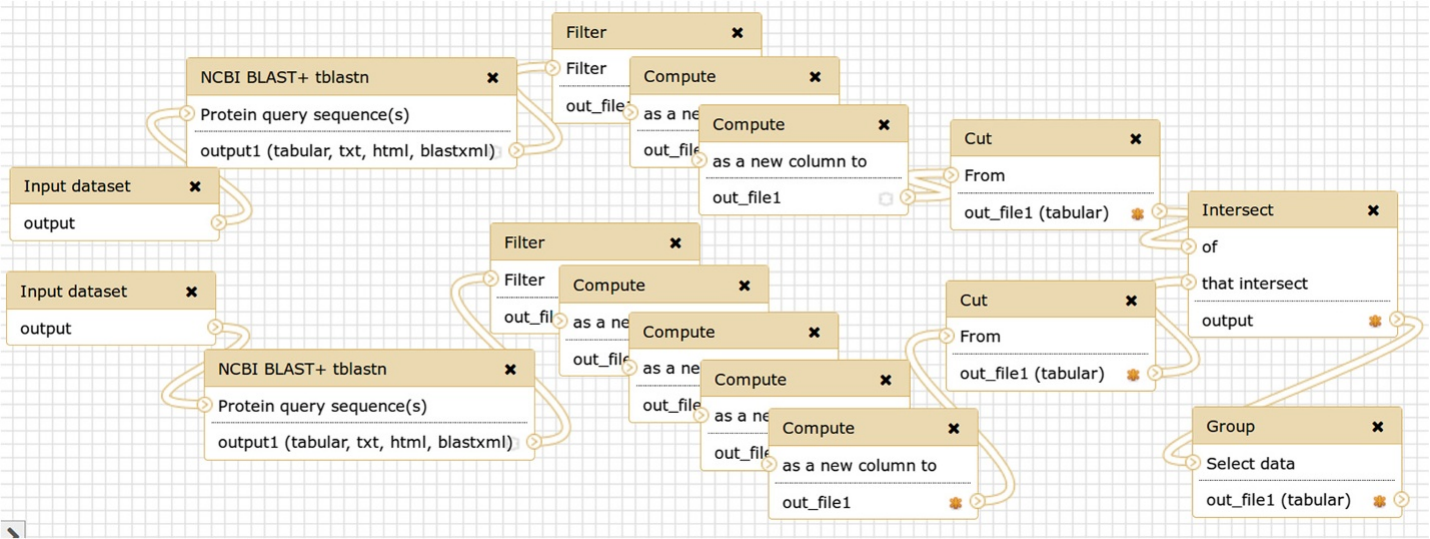
Galaxy workflow for finding gene clusters. Screenshot from the Galaxy Workflow Editor, showing a published example workflow [27] discussed in the Analyses section. Given two protein sequences, regions of a genome of interest are identified that contain tblastn matches to both sequences, which pinpoints candidate gene clusters for further study

For this application a workflow was constructed to query two translated protein sequences against a BLAST nucleotide database for the target genome [27] (Fig. 1). This workflow is available with sample data via the Galaxy Tool Shed [28] and myExperiment [29].

The TBLASTN results are processed with standard Galaxy text manipulation tools to extract the target sequence identifier and the hit start and stop coordinates. The three-column interval format obtained is Browser Extensible Data (BED)-like and the sequence identifier corresponds to the chromosome or contig name. Before intersecting the hit regions, one of them is extended by 10,000 bp upstream and by the same length downstream, by adding and subtracting 10,000 from the start and end coordinates, respectively. The intersect tool works on genomic coordinates, identifying overlapping regions. These regions encode similar proteins to the query sequence and other proteins in close proximity (<10,000 bp). The optional and last step in this example groups and counts all sequence identifiers, returning a list of all identified pairs located nearby and their count.

This approach screens two proteins against all nucleotide sequences from the NCBI nucleotide sequence database (NCBI NT) within hours on our cluster, which leads to the identification of all organisms with an interesting gene structure for further investigation. As usual in Galaxy workflows, every parameter, including the proximity distance, can be changed and additional steps can be easily added. For example, additional filtering to refine the initial BLAST hits, or inclusion of a third query sequence, can be added.

#### Identifying novel proteins

Proteogenomics combines genomic information with mass-spectrometry-derived experimental data for proteomic analysis. To search for evidence of novel proteins, the databases for proteomics search applications are generated from six-frame translations of genomics or transcript sequences or cDNA transcripts. With such large databases, proteomics search applications generate a large number of peptide spectral matches (PSMs). The University of Minnesota developed workflows in Galaxy-P (https://usegalaxyp.org/) to automate proteogenomic analysis [30]. These workflows use the NCBI BLAST+ wrappers to compare the PSM peptides to known proteins to filter the PSM list for those that are more likely to be novel. An additional protein-protein BLAST (BLASTP) wrapper was deployed in Galaxy-P to use the remote search option of BLASTP to perform taxon-specific searches on NCBI servers.

#### Implementation

Despite its maturity, the Galaxy platform has continued to evolve rapidly, especially in the area of tool definition and distribution. The Galaxy Tool Shed [9], published in 2014, enables anyone hosting a Galaxy instance to install tools and defined dependencies with a few clicks right from the Galaxy web application itself. The NCBI BLAST+ tools described here were among the first tools migrated to the Galaxy Tool Shed and have served as drivers of Tool Shed features and representative examples of how easy it can be to deploy very powerful tools using Galaxy.

The Galaxy BLAST+ wrappers are developed as an open-source project using the distributed version control system Git. We use the hosting service provided by GitHub, Inc., which has become the hub of a growing software development ecosystem. One particular example of this is the continuous integration service travis-ci.org offered by Travis CI GmbH. Although complex to set up, every time our source code is updated on GitHub, Travis CI automatically creates a Linux virtual machine and installs BLAST+, the latest Galaxy code and our wrappers - whose functional tests are then run [31]. This integration provides us prompt feedback, through which many errors can be caught and dealt with before releasing a new version via the Galaxy Tool Shed. Furthermore, the BLAST+ wrapper tests have been used by the Galaxy development team when working on the Galaxy test framework.

One of the core concepts in Galaxy is that each dataset has a specified datatype or file format, such as FASTA format sequences or various FASTQ encodings [32]. Each Galaxy tool normally accepts only specific datatypes as input and will mark its output files with the appropriate datatype. We defined a set of datatypes for BLAST ASN.1 files, BLAST XML and the different BLAST database types (see Table 3). Simple datatypes can be defined by subclassing already existing datatypes. In general, additional Python code is required, such as defining a sniff function for auto-detection of the datatype when loading files into Galaxy.

Galaxy also supports simple job splitting, which works at the datatype level, with input datatypes (such as FASTA) needing to provide a split method and output datatypes (such as tabular or BLAST XML) needing to provide a merge method. If this job splitting is enabled, BLAST searches are automatically parallelized by splitting the FASTA query file into chunks and then merging the output BLAST results. This process is done transparently to the user and enables genome-scale BLAST jobs to be spread across a cluster rather than being processed serially, providing a dramatic speedup.

The Galaxy-P project (Minnesota Supercomputing Institute, University of Minnesota) contributed extensions to Galaxy known as tool macros that make it considerably easier to develop and maintain large suites of Galaxy tools by allowing authors to define high-level abstractions describing any aspect of Galaxy’s XML-based tool description language. These abstractions can be combined and shared across various tools in a suite. In wrapping the NCBI+ BLAST tool suite we have made heavy use of macros to avoid the duplication of common parameters, command line arguments and even help text. In addition to removing hundreds of lines of XML, this approach helps with consistency and maintenance, as many changes need only be made once to the macro definition.

Although the Galaxy Tool Shed has greatly simplified installation of additional tools to an existing Galaxy server, doing this installation ‘by hand’ remains time-consuming and reproducibility suffers. However, this process can be scripted, which is useful for automated testing (as in our Travis CI setup outlined above) but vital for large-scale deployment. In a similar vein to the Galaxy CloudMan project [14] for automated creation of complete virtual machine images running Galaxy, we used the virtual containers technology from Docker, Inc. for testing and deployment of a Galaxy server complete with additions such as the BLAST+ tools. The Galaxy BLAST Docker Image (see Availability and Requirements section) offers a complete Galaxy instance with file transfer protocol (FTP) server, job scheduler and BLAST wrappers [33]. Once Docker Image is installed, the command ‘docker run -p 8080:80 bgruening/galaxy-blast’ will download the image and start a BLAST-enabled Galaxy instance on port 8080. Note that the Docker Image does not currently automate installation of any BLAST databases.

One area that remains a burden for the Galaxy administrator is the provision of local copies of BLAST databases (external to Galaxy), such as in-house unpublished datasets, or the main NCBI BLAST databases [34]. The locations of these databases (which can be used outside of Galaxy) are listed in simple tabular configuration files (blastdb*.loc), which store a unique identifier key (recorded in Galaxy), a description (shown to the Galaxy user) and the file path to the database (which can be updated if required, for example owing to changes in local storage architecture). In future work we hope to use the Galaxy Data Manager Framework [35] to facilitate the provision of BLAST databases.

### Discussion

Over the past few decades the BLAST suite has grown, with improvements such as gapped searches [36] and additional functionality such as Position-Specific Iterated BLAST (PSI-BLAST) [36, 37] and protein-domain searches with Reverse Position-Specific BLAST (RPS-BLAST) [38]. These Position-Specific Score Matrix (PSSM)-based tools underpin the NCBI Conserved Domain Database (CDD) and the associated web-based Conserved Domain Search service (CD-Search) [38, 39]. More recently, the NCBI BLAST team undertook an ambitious rewrite of the BLAST tool suite, converting the existing ‘legacy’ code base, which was written in the C programming language, to the C++ language. The new version was dubbed BLAST+ [16].

The expansion of the Galaxy wrappers for BLAST+ has followed a similar course. The initial wrappers focused on the five core tools (BLASTP, BLASTN, BLASTX, TBLASTN and TBLASTX) and did not allow the creation of custom BLAST databases. Gradually, the scope and contributor base of the project has expanded (Tables 1 and 3), particularly since our publication of genome and protein annotation tools [10], and was also supported by the move to a dedicated source code repository on GitHub. This shift to a distributed international team effort followed discussions, both online and in person at the Galaxy Community Conference 2013, and reflects the broad usage of the BLAST+ tools within the Galaxy community.

Future work will include additional wrappers for the remaining or new BLAST+ command line tools, exposing additional command line options via the Galaxy interface, and additional output file formats. Developments within Galaxy will also allow new functionality. For example, we hope to build on the Galaxy Visual Analysis Framework [40] to offer graphical representation of BLAST results within Galaxy, such as that offered by the NCBI web service. Similarly, managing local BLAST databases could be facilitated using the Data Manager Framework [35].

By their nature, the Galaxy *.loc files and associated external datasets (such as NCBI BLAST databases) impose an administrative overhead and limitations on reproducibility. One problem is that versioning of external datasets requires a copy of each revision be maintained with its own entry in Galaxy’s corresponding *.loc file. In the case of the NCBI BLAST databases, this provenance tracking is hampered by the absence of official versioning. Here a date-stamping approach is possible, for example by keeping quarterly snapshots if local storage allows. However, the more practical and probably more common approach is to have a single live copy of the NCBI BLAST databases, kept up to date automatically with the NCBI-provided Perl scripts or similar. Such setups are often already in place on central computer clusters used for bioinformatics. A second issue with using external datasets in Galaxy is that they undermine sharing of workflows between Galaxy servers, as any referenced external datasets must also be synchronized. At a practical level this synchronization requires consistent naming schemes. For instance, for current versions of the NCBI BLAST databases we recommend that the Galaxy administrator always use the case-sensitive stem of the file name as the key (e.g., use nr in blastdb_p.loc to refer to a current version of the NCBI non-redundant protein sequence database).

Running BLAST+ locally within Galaxy has been particularly useful for multi-query searches and searching against unpublished data, such as draft genomes, as both the local administrator and individual users can create databases. However, the biggest user benefits for data processing come when complete workflows can be run within Galaxy, as in the examples shown.

## Availability and requirements

**Project name:** Galaxy wrappers for NCBI BLAST+ and related BLAST tools

**Project home page:**https://github.com/peterjc/galaxy_blast

**Operating system(s):** Linux (recommended), Mac

**Programming language:** Python

**Other requirements:** Galaxy (and dependencies therein), NCBI BLAST+

**License:** The MIT License

**Any restrictions to use by non-academics:** None

The Galaxy wrappers are also available from the Galaxy Tool Shed (https://toolshed.g2.bx.psu.edu/view/devteam/ncbi_blast_plus) for installation to an existing Galaxy server and as part of Docker Image (https://registry.hub.docker.com/u/bgruening/galaxy-blast/), which provides a Galaxy server with the BLAST+ tools preinstalled.

## Availability of supporting data

The datasets supporting the results of this article are available in the Galaxy BLAST repository, https://github.com/peterjc/galaxy_blast (i.e., sample files used for automated functional testing). A snapshot is also hosted in the *GigaScience* GigaDB repository [41].

## Abbreviations

BLAST: Basic Local Alignment Search Tool
BLASTN: Nucleotide BLAST
BLASTP: Protein BLAST
BLASTX: BLAST for searching protein databases using a translated nucleotide query
FASTA: Text format for biological sequences
FASTQ: Text format for biological sequences with quality scores
NCBI: National Center for Biotechnology Information
PSM: Peptide spectral match
TBLASTN: BLAST for searching translated nucleotide databases using a protein query
TBLASTX: BLAST for searching translated nucleotide databases using a translated nucleotide query
XML: Extensible Markup Language
